# Time to *Staphylococcus aureus* Blood Culture Positivity as a Risk Marker of Infective Endocarditis: A Retrospective Cohort Study

**DOI:** 10.1093/cid/ciae628

**Published:** 2024-12-21

**Authors:** Martin Strömdahl, Karl Hagman, Karolina Hedman, Anna Westman, Magnus Hedenstierna, Johan Ursing

**Affiliations:** Department of Infectious Diseases, Danderyd Hospital, Stockholm, Sweden; Department of Clinical Sciences, Karolinska Institutet, Danderyd Hospital, Stockholm; Department of Infectious Diseases, Sahlgrenska University Hospital; Department of Infectious Diseases, Institute of Biomedicine, Sahlgrenska Academy at University of Gothenburg, Gothenburg, Sweden; Department of Microbiology, Karolinska University Hospital, Stockholm, Sweden; Department of Infectious Diseases, Danderyd Hospital, Stockholm, Sweden; Department of Microbiology, Karolinska University Hospital, Stockholm, Sweden; Department of Infectious Diseases, Danderyd Hospital, Stockholm, Sweden; Department of Clinical Sciences, Karolinska Institutet, Danderyd Hospital, Stockholm; Department of Infectious Diseases, Danderyd Hospital, Stockholm, Sweden; Department of Clinical Sciences, Karolinska Institutet, Danderyd Hospital, Stockholm

**Keywords:** bacteremia, infective endocarditis, *Staphylococcus aureus*, time to blood culture positivity, bloodstream infection

## Abstract

**Background:**

Endocarditis occurs in approximately 10%–15% of patients with *Staphylococcus aureus* bacteremia (SAB). Short time to positivity (TTP) in blood culture flasks has been linked to endocarditis in smaller studies. This study evaluated the association between TTP and endocarditis in SAB in a large cohort.

**Methods:**

Adult patients with ≥1 *S. aureus* positive blood culture treated at a tertiary-level, 500-bed hospital in Stockholm, Sweden between 2011–2021 were retrospectively identified. The primary outcome was the presence of infective endocarditis.

**Results:**

A total of 1703 episodes of SAB (23/1703 methicillin-resistant) in 1610 patients were included. Median age was 75 (interquartile range [IQR], 63–84) years and median Charlson comorbidity index score was 2 (IQR, 1–3). Echocardiography was performed in 1102/1703 (65%). Thirty-day mortality was 406/1703 (24%) and endocarditis was found in 154/1703 (9%). Median TTP was shorter in patients with endocarditis (9 [IQR, 7–12] hours) compared to patients without endocarditis (13 [interquartile range, 10–18] hours; *P* < .001). The risk of endocarditis decreased with 11% per hour (odds ratio [OR], 0.89 [95% confidence interval {CI}, .54–.92]; *P* < .001) in a univariate analysis using TTP as a continuous variable. In multivariate analysis, TTP <13 hours (the median) was independently associated with endocarditis (OR, 3.59 [95% CI, 2.35–5.3]; *P* < .001). The negative predictive value of TTP >13 hours for endocarditis was 96% (95% CI, 95%–97%).

**Conclusions:**

Short TTP was associated with endocarditis. The negative predictive value of >95% suggests that TTP >13 hours can be used to risk-stratify patients with SAB.

## Teaser

Short time to blood culture positivity (TTP) was an independent risk factor of infective endocarditis in this large (N = 1703) *Staphylococcus aureus* bacteremia cohort. TTP is useful for endocarditis risk stratification.

The incidence of *Staphylococcus aureus* bacteremia (SAB) is 10–30 per 100 000 person-years [1]. The 30-day mortality remains around 20% despite advances in medical treatment [2–5]. Local foci of infection are established in almost half of patients, and endocarditis occurs in 10%–15% of patients with SAB [5–8]. Established risk factors of endocarditis in SAB include hemodialysis, unknown focus of infection, presence of implanted prosthetic heart valves, and growth in a blood culture taken after 2–4 days of effective antibiotic treatment [6–11]. The risk of endocarditis and other deep-seated foci of infection are also higher in community-acquired compared to nosocomial SAB [8, 12].

Time to positivity (TTP) is an indirect measure of the *S. aureus* inoculate in a blood culture flask and is defined as the time from start of incubation to positive signal of bacterial growth. TTP has mostly been investigated in catheter-associated bloodstream infections. More recently, TTP has also been evaluated as a marker of complicated disease or death. TTP longer than 11–14 hours was associated with reduced risk of endocarditis in smaller SAB studies, and TTP exceeding 13 hours excluded endocarditis in a cohort of 465 SAB cases [13–16]. Greater bacterial load in blood has recently been linked to endocarditis, supporting the association between fast TTP and endocarditis [17, 18]. In 1 study (N = 587), the risk of endocarditis increased with both short (<10 hours) and long (>18 hours) TTP compared to intermediate (10–18 hours) TTP [19].

Current guidelines recommend a minimum of 2 weeks of intravenous (IV) antibiotics and preferably transesophageal echocardiography (TEE) to detect endocarditis for all patients with SAB [9, 20]. Clinical experience and recently published studies indicate that early oral switch may be possible for uncomplicated SAB [21–24]. Early identification of patients with and without a high risk of endocarditis may thus enable faster initiation of correct endocarditis therapy, reduce the need for TEE, and shorten IV treatment durations and hospital stays. This study aimed to evaluate the association between TTP and infective endocarditis and assess the ability of prolonged TTP to exclude endocarditis in a retrospective cohort of patients with SAB.

## METHODS

### Study Design and Population

This was a retrospective single-center study of patients with SAB treated at a tertiary-level 500-bed hospital (Danderyd Hospital) in Stockholm, Sweden. The population in the hospital catchment area is approximately 650 000 people. Microbiology Department records were searched to identify patients with growth of *S. aureus* in blood cultures collected at Danderyd Hospital between 1 January 2011 and 31 December 2021. Patients ≥18 years old with growth of *S. aureus* in ≥1 blood culture bottle were included in the study. Exclusion criteria were transfer to another hospital for definitive care (as this precluded data extraction) or SAB relapse, defined as <30 days since completion of treatment for previous SAB.

Data on demographics, comorbidities, treatments, examinations, and outcomes such as presence of endocarditis, surgery, mortality, and readmission were manually extracted from electronic medical records (TakeCare, CompuGroup Medical Sweden AB).

Blood cultures were transported to the Department of Microbiology at Karolinska University Hospital, located 7 km from Danderyd Hospital, throughout the study. The routines for managing blood cultures changed over time and were as follows: Between January 2011 and December 2018, blood cultures were sent 6 times daily on normal working days and 3 times daily during weekends and holidays without any prior incubation. Blood cultures were stored in room temperature while awaiting transport. Upon arrival at the Department of Microbiology, cultures were incubated using BacT/ALERT 3D (bioMérieux), 8 Am to 8 Pm during weekdays and 8 Am to 4 Pm on weekends and holidays. From December 2018, the flasks were immediately incubated at Danderyd Hospital (whatever the time of day) before transportation to the Department of Microbiology. From this time point, both sites used BacT/ALERT Virtuo (bioMérieux) cabinets. Species identification after October 2012 was made using matrix-assisted laser desorption/ionization–time of flight and prior to that using a combination of VITEK-1, DNase test, and Staphaurex agglutination test. Methicillin-resistant *S. aureus* (MRSA) was confirmed by in-house polymerase chain reaction detecting *nuc* and *mecA* genes between 2011 and September 2019 and after that using the Easyplex MRSA plus assay (AmplexDiagnostics GmbH).

### Primary Outcome

The primary outcome was the presence of infective endocarditis.

### Definitions and Variables

Fastest time to growth in the initial blood culture set was recorded as TTP. Charlson comorbidity index (CCI) score was calculated [25]. Diagnosis of endocarditis was made by the treating physician according to the Swedish Society for Infectious Disease guidelines on infective endocarditis, which is based on the modified Duke criteria [26]. Mode of *S. aureus* acquisition was stratified into 3 categories as described by Friedman et al; in brief, these were (1) community-acquired if the infection was diagnosed <48 hours after admission to a hospital; (2) healthcare-associated if the patient was residing in a nursing home, received IV therapy at home, received IV chemotherapy during the last 30 days, received hemodialysis therapy, or was treated in an acute care hospital during the last 90 days; or (3) nosocomial if the infection was diagnosed >48 hours after hospitalization [27]. “Community-onset” was used to describe infections that were either community-acquired or healthcare-associated. Persistent bacteremia was defined as blood cultures positive for *S. aureus* taken 24–96 hours after the original blood cultures as recommended by the Infectious Diseases Society of America [9]. Relapse was defined as growth of *S. aureus* in blood cultures within 30 days of the last day of antibiotic therapy for the original episode of bacteremia.

### Ethical Considerations

Ethical permission was granted from the Swedish Ethical Review Authority in Linköping, Sweden (registration number 2022-01212-01).

### Statistical Analysis

Continuous variables are presented as median (interquartile range [IQR]) and categorical variables as number (percentage). Univariate and multivariate binominal logistic regression models were used to identify the ability of TTP from initial blood cultures to predict endocarditis. TTP and previously reported endocarditis risk factors were independent variables and infective endocarditis was the dependent variable in the regression analyses. Variables with a *P* value <.1 in the univariate analysis were included in the multivariate analysis, and relative risk was expressed as odds ratio (OR). Sensitivity, specificity, and receiver operating characteristic (ROC) curve of TTP to identify endocarditis, and their 95% confidence intervals (CIs), when applicable, were calculated. Fisher exact test was used to calculate *P* values in the sensitivity and specificity analysis. A *P* value <.05 was considered significant. Positive predictive values (PPVs) and negative predictive values (NPVs) were calculated from 2 × 2 contingency tables without accounting for prevalence. The distribution of TTP in 2011–2018 versus 2019–2021 was compared using the Mann-Whitney *U* test. Youden index was calculated to identify the optimal TTP with regard to both specificity and sensitivity. However, the cohort was analyzed after stratification according to the 25th, 50th (median), and 75th percentiles of TTP for comparison with previous studies and because we aimed to describe an optimal NPV as opposed to the best fit. Analyses were performed using IBM SPSS Statistics version 26 software (IBM Corporation, Armonk, New York). Boxplot figures and the ROC curve figure were created using the Julius online tool (www.julius.ai). In the figures, whiskers represent the 5th and 95th percentiles while outliers are not shown.

## RESULTS

The demographics of the cohort are presented in Table 1. A total of 1776 episodes of SAB in patients ≥18 years old between 2011 and 2021 were identified. Thirty episodes were defined as relapses and thus excluded, and 43 were excluded because they were transferred to another hospital. Among the 1703 episodes of SAB (23/1703 MRSA) in 1610 patients who were included, median age was 75 (IQR, 63–84) years and median CCI score was 2 (IQR, 1–3). Echocardiography was performed in 65% (1102/1703) of the whole cohort and in 70% (1091/1557) of patients who survived >96 hours. Thirty-day mortality was 406 of 1703 (24%). TTP was available in all cases but 1 (n = 1702). Median TTP was 13 (IQR, 10–18) hours. Follow-up cultures were taken in 496 of 1703 (29%) cases and persistent bacteremia was found in 333 of 496 (67%). The percentage of patients with community-acquired, healthcare-associated, and hospital-acquired SAB was 29.5% (n = 502/1703), 41% (n = 698/1703), and 29.5% (n = 503/1703), respectively.

**Table 1.** Demographic and Clinical Characteristics of Patients With *Staphylococcus aureus* Bacteremia According to Mode of Acquisition

Characteristic	All Episodes (N = 1703)	Community-Acquired (n = 502)	Healthcare-Associated (n = 698)	Hospital-Acquired (n = 503)
Male sex	1107 (65)	334 (67)	465 (67)	308 (61)
Age, y, median (IQR)	75 (63–84)	73 (59–83)	77 (67–86)	75 (63–83)
Pacemaker and/or ICD	197 (12)	44 (9)	91 (13)	62 (13)
Prosthetic heart valve	69 (4)	13 (3)	33 (5)	23 (5)
Vascular graft	12 (1)	6 (1)	2 (<1)	4 (1)
Arthroplasty	120 (7)	40 (8)	48 (7)	32 (6)
Intravenous drug use	24 (1)	19 (4)	5 (1)	0
Hemodialysis	66 (4)	0	58 (8)	8 (2)
CCI score, median (IQR)	2 (1–3)	1 (0–2)	2 (1–4)	2 (1–3)
Diabetes mellitus	484 (28)	138 (28)	206 (30)	140 (28)
Myocardial infarction	210 (12)	41 (8)	103 (15)	66 (13)
Congestive heart failure	461 (27)	84 (17)	203 (29)	174 (35)
Stroke or TIA	283 (17)	55 (11)	135 (20)	93 (19)
Dementia	144 (9)	18 (4)	105 (15)	21 (4)
Solid tumor	131 (8)	29 (6)	61 (9)	41 (8)
Metastatic cancer	78 (5)	14 (3)	42 (6)	22 (4)
MRSA bacteremia	23 (1)	7 (1)	10 (1)	6 (1)
Time to positivity, h, median (IQR)	13 (9.8–17.5)	12.8 (9.1–17)	13 (9.6–18)	12.8 (10–17)
1st quartile (2–9.7 h)	424 (25)	138 (28)	180^a^ (26)	106 (21)
2nd quartile (9.8–13 h)	420 (25)	119 (25)	150^a^ (22)	151 (30)
3rd quartile (13.1–17.3 h)	430 (25)	127 (25)	176^a^ (25)	127 (25)
4th quartile (17.4.1–110.2 h)	428 (25)	118 (24)	191^a^ (27)	119 (24)
Follow-up culture within 24–96 h	496 (29)	156 (31)	173 (25)	167 (33)
Persistent bacteremia	333/496 (67)	108/156 (69)	120/173 (69)	105/167 (63)
TTE and/or TEE	1102 (65)	363 (72)	410 (59)	329 (65)
Infective endocarditis	154 (9)	68 (14)	54 (8)	32 (6)
Readmission within 30 d	294 (17)	79 (16)	121 (17)	94 (19)
Related to SAB	56 (3)	21 (4)	20 (3)	15 (3)
Mortality within 96 h	146 (9)	27 (5)	76 (11)	43 (9)
30-day mortality	406 (24)	79 (16)	185 (27)	142 (28)

Data are presented as No. (%) unless otherwise indicated.

Abbreviations: CCI, Charlson comorbidity index; ICD, implantable cardioverter-defibrillator; IQR, interquartile range; MRSA, methicillin-resistant *Staphylococcus aureus*; SAB, *Staphylococcus aureus* bacteremia; TEE, transesophageal echocardiography; TIA, transient ischemic attack; TTE, transthoracic echocardiography.

^a^Missing time to positivity data for 1 case.

### TTP as a Risk Factor for Endocarditis

**Figure 1.**
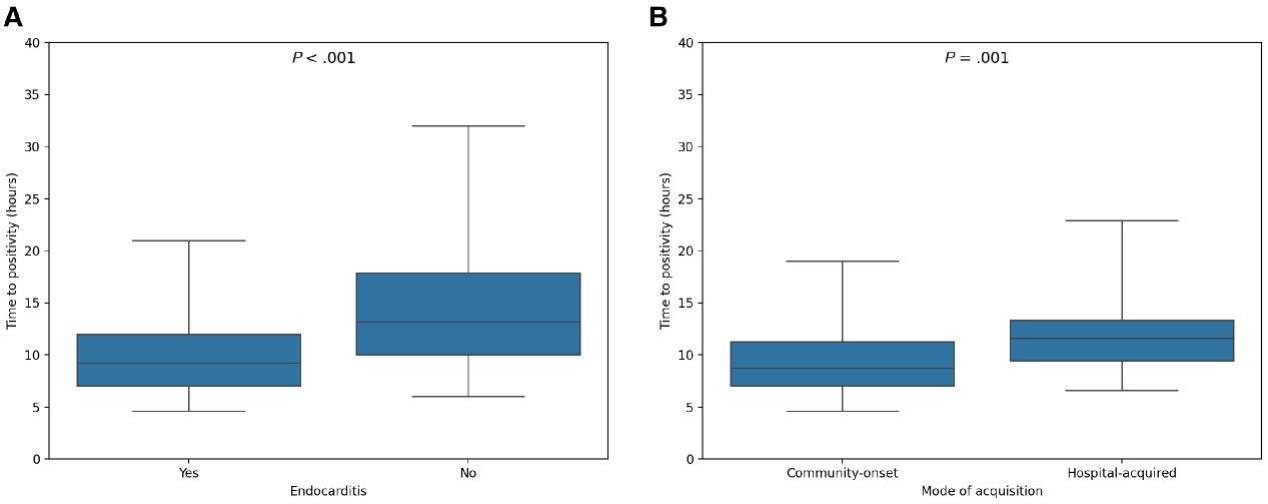
*A*, Boxplot of time to positivity in episodes with (n = 154) and without (n = 1549) endocarditis. *B*, Boxplot of time to positivity in episodes with endocarditis with community-onset (n = 122) or hospital-acquired (n = 32) *Staphylococcus aureus* bacteremia.

Endocarditis was present in 154 of 1703 (9%) episodes of SAB. Median TTP was shorter in patients with endocarditis (9 [IQR, 7–12] hours) compared to patients without endocarditis (13 [IQR, 10–18] hours; *P* < .001; Figure 1*A*). In a univariate analysis using TTP as a continuous variable, the risk of endocarditis decreased with 11% per hour (OR, 0.89 [95% CI, .54–.92]; *P* < .001). In further analyses, TTP shorter than the median value (13 hours) was tested as a risk factor for endocarditis. Presence of a pacemaker and/or implantable cardioverter-defibrillator (ICD), prosthetic heart valve, vascular graft, IV drug use, community acquisition, persistent bacteremia, and TTP <13 hours were associated with endocarditis in univariate analyses (Table 2). In the multivariate analysis, the same variables including TTP <13 hours (OR, 3.59 [95% CI, 2.35–5.3]; *P* < .001) were independently associated with endocarditis (Table 2). Follow-up cultures were not included in the multivariate analysis as they were not done in 1207 episodes. The PPVs for endocarditis were ≤20% when using the 25th, 50^th^, and 75th percentile as cutoffs (Table 3). The PPVs for endocarditis improved slightly when hospital-acquired infections were excluded and hospital-associated and community-acquired infections were pooled into a community-onset group (n = 1199) (Table 3). In line with this, median TTP was significantly faster (9 [IQR, 7–11] hours) in patients with endocarditis classified as community-onset (n = 122) compared to hospital-acquired (n = 32) cases of endocarditis (12 [IQR, 9–14] hours; *P* = .001; Figure 1*B*).

**Table 2.** Risk Factors of Infective Endocarditis in *Staphylococcus aureus* Bacteremia Episodes

Risk Factor	Univariate Analysis			Multivariate Analysis		
	OR	(95% CI)	*P* Value	OR	(95% CI)	*P* Value
Male sex	0.91	(.64–1.28)	.58	…	…	
Age, y	1	(.99–1.01)	.3	…	…	
Pacemaker and/or ICD	2.1	(1.37–3.21)	.001	1.96	(1.25–3.1)	.001
Prosthetic heart valve	5.33	(3.11–9.11)	<.001	4.22	(2.36–7.52)	<.001
Vascular graft	7.39	(2.32–23–58)	.001	4.26	(1.23–14.76)	<.001
Arthroplasty	1.24	(.68–2.27)	.48	…	…	
Intravenous drug use	7.61	(3.32–17.45)	<.001	7.57	(3.08–18.58)	<.001
Hemodialysis	1.85	(.93–3.71)	.08	2.25	(1.06–4.8)	.08
CCI score	0.97	(.89–1.06)	.51	…	…	
Place of acquisition						
Hospital-acquired	Ref.	…		Ref.	…	
Healthcare-associated	1.23	(.79–1.94)	.36	1.18	(.73–1.91)	.5
Community-acquired	2.31	(1.49–3.58)	<.001	2.36	(1.48–3.77)	<.001
TTP <13 h	4.02	(2.71–5.96)	<.001	3.59	(2.35–5.3)	<.001
Persistent bacteremia 24–96 h^a^	2.4	(1.14–5.07)	.02	…	…	

Variables from univariate analysis with *P* < .1 were included in the multivariate analysis.

Abbreviations: CCI, Charlson comorbidity index; CI, confidence interval; ICD, implantable cardioverter-defibrillator; OR, odds ratio; Ref, reference; TTP, time to positivity.

^a^Data only available for 496 of 1703 cases of bacteremia; this variable was therefore not included in the multivariate analysis.

**Table 3.** Positive and Negative Predictive Values and Numbers of Episodes With and Without Endocarditis Stratified by Place of Acquisition and Time to Positivity in Hours

Time to Positivity, h	Endocarditis, No. of Episodes	No Endocarditis, No. of Episodes	PPV, % (95% CI)	NPV, % (95% CI)
Whole cohort (N **=** 1702)				
TTP **<**10 (25th percentile)	84	334	20 (17–23)	95 (94–95)
TTP >10	70	1208		
TTP <13 (median)	120	724	14 (13–15)	96 (95–97)
TTP >13	34	824		
TTP <17 (75th percentile)	138	1136	11 (10–11)	96 (94–98)
TTP >17	16	421		
Community-onset (n = 1199)				
TTP <10	75	243	24 (21–28)	95 (93–96)
TTP >10	47	834		
TTP <13	100	487	17 (16–19)	96 (95–98)
TTP >13	22	590		
TTP <17	122	778	14 (13–14)	97 (94–98)
TTP >17	10	299		
Community-acquired (n = 502)				
TTP <10	50	92	35 (30–41)	95 (93–97)
TTP >10	18	342		
TTP <13	60	197	23 (21–26)	97 (94–98)
TTP >13	8	237		
TTP <17	64	320	17 (16–18)	97 (92–99)
TTP >17	4	114		
Healthcare-associated (n = 697)				
TTP <10	27	163	14 (11–18)	95 (93–96)
TTP >10	27	480		
TTP <13	40	290	12 (10–14)	96 (94–98)
TTP >13	14	353		
TTP <17	48	458	9 (9–10)	97 (93–99)
TTP >17	6	185		
Hospital-acquired (n = 503)				
TTP <10	9	97	8 (5–14)	95 (93–95)
TTP >10	23	374		
TTP <13	20	237	8 (6–10)	95 (93–97)
TTP >13	12	234		
TTP <17	26	358	7 (6–8)	95 (90–98)
TTP >17	6	113		

Abbreviations: CI, confidence interval; NPV, negative predictive value; PPV, positive predictive value; TTP, time to positivity.

### TTP to Exclude Endocarditis

**Figure 2.**
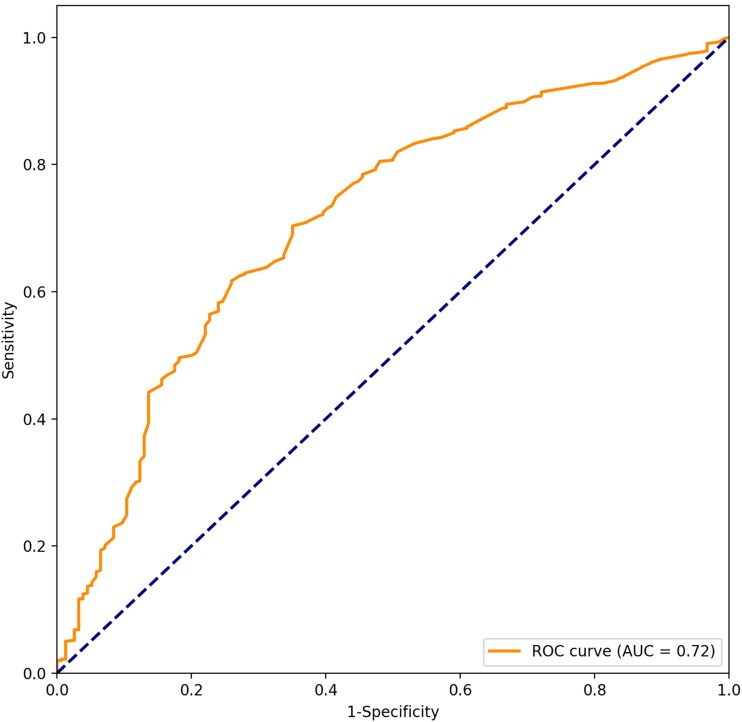
Receiver operating characteristic (ROC) curves of the ability of time to positivity to differentiate *Staphylococcus aureus* bacteremia episodes with and without infective endocarditis. Area under the curve (AUC) was 0.72 (95% confidence interval, .67–.76; *P* < .001).

The ROC area under the curve (AUC) for TTP to discriminate endocarditis from cases without endocarditis was 0.72 (95% CI, .67–.76; *P* < .001) for the whole cohort (Figure 2), and the Youden index was 11.85 hours. The NPVs were ≥95% for the 25th (10 hours), 50th (13 hours), and 75th (17 hours) percentiles of TTP as cutoffs for the whole cohort (Table 3).

### Effect of Site of SAB Acquisition on the Ability of TTP to Exclude Endocarditis

In patients with community-acquired, healthcare-associated, and hospital-acquired SAB, the AUC for TTP to exclude endocarditis was 0.81 (95% CI, .75–.86), 0.68 (95% CI, .61–.75), and 0.58 (95% CI, .48–.69), respectively. TTP demonstrated an AUC for excluding endocarditis of 0.75 (95% CI, .70–.79) when community-acquired and healthcare-associated infections were pooled in a community-onset SAB group. The NPVs were ≥95% using either TTP cutoff regardless of site of SAB acquisition (Table 3).

### Effect of Blood Culture Handling on TTP

**Figure 3.**
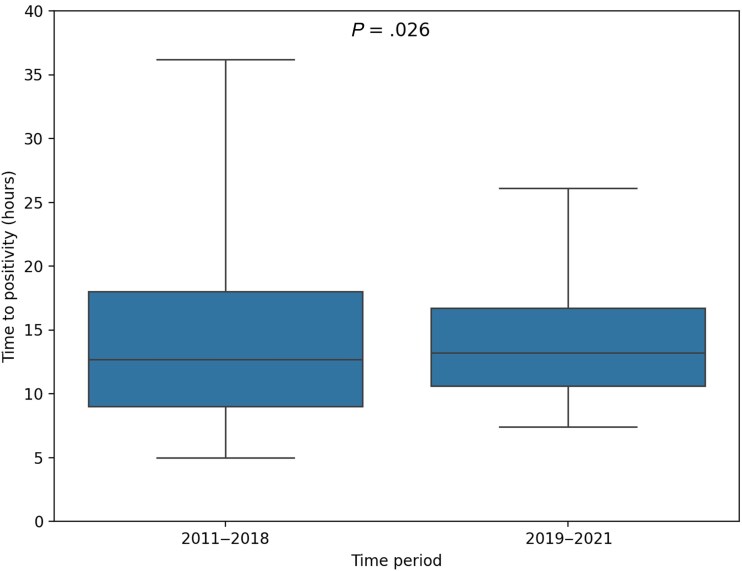
Boxplot of time to positivity in *Staphylococcus aureus* bacteremia episodes from 2011 to 2018 and 2019 to 2021, respectively.

Two groups were created by splitting the cohort using the change in blood culture logistics in December 2018 as a dividing point: the transport before incubation (from 1 January 2011 to 31 December 2018, n = 1029) and the immediate incubation (1 January 2019 to 31 December 2021, n = 674) groups. Median TTP for the 2011–2018 and the 2019–2021 groups was 13 (IQR, 9–18) and 13 (IQR, 11–17) hours, respectively. The 5th and 95th percentiles were 5 and 38 hours for the 2011–2018 group and 7 and 26 for the 2019–2021 group (*P* = .026; Figure 3). In the 2019–2021 group, 10 of 354 (3%) patients with TTP >13 hours were diagnosed with endocarditis compared to 24 of 504 (5%) patients in the 2011–2018 group with TTP >13 hours (*P* = .16; Supplementary Table 1).

Ability of TTP to Exclude Endocarditis When Blood Culture Handling Was Optimized in Community-Acquired or Community-Onset Infection

Restricting the analysis to the site of acquisition associated with the highest risk of endocarditis (community-acquired SAB) when sampling was optimized (years 2019 and 2021) (n = 212, 29 cases of endocarditis) produced an AUC of 0.87 (95% CI, .82–.93) and NPVs of 98% (95% CI, 98%–100% [2/113]) and 100% (95% CI, 93%–100% [0/48]) when the TTP cutoffs were 13 and 17 hours, respectively (Supplementary Table 1).

Analyzing community-onset SAB during the same period (n = 452, 46 cases of endocarditis), the AUC to identify endocarditis was 0.82 (95% CI, .76–.87) and NPVs were 98% (95% CI, 95%–99% [5/239]; *P* < .001) and 99% (95% CI, 94%–100% [1/102]) for TTP cutoffs of 13 and 17 hours, respectively (Supplementary Table 1).

## DISCUSSION

In this retrospective cohort of 1703 episodes of SAB including 154 cases of endocarditis, shorter TTP (faster than the median TTP of 13 hours) was associated with an increased risk of endocarditis. The risk increase (OR, 3.59) in the multivariate analysis was of similar magnitude as reported in previous studies [19, 28]. The risk increase was also of comparable magnitude to the risk associated with established factors, such as hemodialysis, persistent bacteremia, the presence of prosthetic heart valves or pacemakers, and community acquisition [7, 8, 12, 29]. These data corroborate previous studies and indicate that every effort should be made to confirm or exclude endocarditis in SAB patients with a fast TTP.

As information on TTP is immediately available, we assessed its usefulness to identify a subgroup with low risk of endocarditis. The NPV of the 50th and 75th percentile TTP of 13 and 17 hours was 96% (95% CI, 95%–97% and 94%–98%, respectively). The likelihood of having endocarditis was accordingly very small with either cutoff and raising the cutoff did not improve the predictive value. These data are in line with earlier studies of SAB that found NPVs close to 100% with TTP cutoffs of 11–14 hours. As reported in most other studies, there is no clinically relevant absolute cutoff and a handful cases of endocarditis will have a longer time to growth [13, 14].

During the 11-year period examined, 34 of 154 cases of endocarditis had a TTP >13 hours. Nine cases had a pacemaker/ICD or prosthetic valves, suggesting it was indicated to examine them with TEE irrespective of TTP. Choosing the 75th percentile cutoff of 17 hours misclassified 16 endocarditis cases, 5 of which had a pacemaker, ICD, or prosthetic valves (Supplementary Table 2). Thus, even after excluding patients with other obvious reasons for doing a TEE, a clinically useful TTP cutoff to rule out endocarditis could not be found.

The number of misclassified patients decreased further when incorporating the setting of acquisition. Hospital-acquired infections are usually discovered on the first day of fever, when endocarditis has had less time to develop, whereas complications are more likely to already be established upon admission in community-acquired infections. Hence, TTP in blood cultures collected within hours of fever debut is not necessarily comparable to that of blood cultures collected in community-onset infections. In support of this reasoning, we found that the median TTP was significantly slower in hospital-acquired endocarditis cases, the AUC of TTP to exclude endocarditis in the hospital-acquired group was close to 0.5, and excluding hospital-acquired infections from the analysis increased the NPV of TTP <13 hours slightly. When restricting the analysis to community-acquired SAB during 2019–2021 (n = 212), all cases of endocarditis had a TTP of <17 hours (NPV, 100% [95% CI, 93%–100%]; *P* < .001). However, with declining number of patients, the 95% CI correspondingly increased, again suggesting that an absolute cutoff is unlikely.

The present cohort provided a unique opportunity to compare TTP before and after the introduction of immediate incubation. A comparison of TTP distribution over time indicated that the extremes of TTP (Figure 3) approached each other after the shift to around-the-clock incubation. This suggests that TTP will vary depending on transportation and incubation routines for blood cultures in different healthcare settings. Despite immediate incubation during the latter study period, endocarditis could not be entirely ruled out using the median or 75th percentile of TTP (Supplementary Table 1). Consequently, it seems reasonable to remain aware of the limitations of TTP and to use the information it provides together with the clinical context rather than to strive for a specific cutoff time. When the clinical suspicion of endocarditis is low, the response to therapy is rapid, no cardiac devices or prosthetic valves are present, and the TTP is above the median, it might be sufficient to use transthoracic echocardiography solely for cardiac evaluation.

Earlier studies have shown that persistent bacteremia provides information on the risk of complicated SAB [6, 10, 30]. Current recommendations consequently suggest screening using follow-up cultures [9, 20]. In line with this, persistent growth was a significant risk factor of endocarditis in the univariate model (OR, 2.4 [95% CI, 1.14–5.07]; *P* = .02). TTP might provide comparable information on risk as persistent bacteremia, but since only 29% of the patients in the study were screened with follow-up cultures, we were unable to assess that hypothesis.

The strengths of the present study include its large sample size, that each medical record was examined manually (compared to the use of *International Classification of Diseases* codes or automatized data extraction), the inclusion of blood culture logistics, and the use of unique personal identification numbers in Sweden that enable accurate follow-up of relapses and death after discharge. A number of limitations exist: the inherent flaws due to the retrospective and single-center design of the study, that follow-up cultures were not universally done, that time to achieve source control was not recorded, that blood culture volume and whether antibiotics were given prior to its collection were not recorded, and that time from collection of blood cultures to incubation in cabinets was not described. Moreover, only 65% of the patients were examined with echocardiography, which is similar to previously described SAB cohorts and partly due to the substantial early mortality (146/1703 [9%] died during the first 96 hours) [8, 31, 32].

In conclusion, short TTP was associated with increased risk and long TTP was associated with decreased risk of endocarditis in a retrospective cohort of 1703 episodes of SAB. The NPV of TTP over the median of 13 hours was 96% for the whole cohort and 97% for patients in a subset of community-onset infections. TTP is thus a rapidly available marker that contributes to risk stratification in SAB. The data also indicated that immediate incubation of blood cultures increased the predictive value of TTP.

## Supplementary Material

ciae628_Supplementary_Data

## Supplementary Data

Supplementary materials are available at *Clinical Infectious Diseases* online. Consisting of data provided by the authors to benefit the reader, the posted materials are not copyedited and are the sole responsibility of the authors, so questions or comments should be addressed to the corresponding author.

## Notes

***Author Contributions.*** A. W. and K. He. provided microbiological data and details on microbiological methods used during the study period. M. S. extracted data from medical records. M. S., K. Ha., J. U., and M. H. contributed to the data analysis. M. S. and J. U. drafted the manuscript. All authors contributed to data interpretation, critically revised the drafted manuscript, and approved the submitted manuscript. M. S. and J. U. accept full responsibility for the conduct of the study, had full access to all the data, and controlled the decision to publish. M. S. attests that all listed authors meet authorship criteria and that no others meeting the criteria have been omitted.

## References

[1] Tong SY, Davis JS, Eichenberger E, Holland TL, Fowler VG. *Staphylococcus aureus* infections: epidemiology, pathophysiology, clinical manifestations, and management. Clin Microbiol Rev 2015; 28:603–61. 26016486 10.1128/CMR.00134-14 PMC4451395

[2] Bai AD, Lo CKL, Komorowski AS, et al *Staphylococcus aureus* bacteraemia mortality: a systematic review and meta-analysis. Clin Microbiol Infect 2022; 28:1076–84. 35339678 10.1016/j.cmi.2022.03.015

[3] van Hal SJ, Jensen SO, Vaska VL, Espedido BA, Paterson DL, Gosbell IB. Predictors of mortality in *Staphylococcus aureus* bacteremia. Clin Microbiol Rev 2012; 25:362–86. 22491776 10.1128/CMR.05022-11 PMC3346297

[4] Mathé P, Göpel S, Hornuss D, et al Increasing numbers and complexity of *Staphylococcus aureus* bloodstream infection-14 years of prospective evaluation at a German tertiary care centre with multi-centre validation of findings. Clin Microbiol Infect 2023; 29:1197.e9–15. 10.1016/j.cmi.2023.05.031 37277092

[5] Souli M, Ruffin F, Choi S-H, et al Changing characteristics of *Staphylococcus aureus* bacteremia: results from a 21-year, prospective, longitudinal study. Clin Infect Dis 2019; 69:1868–77. 31001618 10.1093/cid/ciz112 PMC6853684

[6] Fowler VG, Olsen MK, Corey GR, et al Clinical identifiers of complicated *Staphylococcus aureus* bacteremia. Arch Intern Med 2003; 163:2066–72. 14504120 10.1001/archinte.163.17.2066

[7] Chang FY, MacDonald BB, Peacock JE, et al A prospective multicenter study of *Staphylococcus aureus* bacteremia: incidence of endocarditis, risk factors for mortality, and clinical impact of methicillin resistance. Medicine (Baltimore) 2003; 82:322–32. 14530781 10.1097/01.md.0000091185.93122.40

[8] Tubiana S, Duval X, Alla F, et al The VIRSTA score, a prediction score to estimate risk of infective endocarditis and determine priority for echocardiography in patients with *Staphylococcus aureus* bacteremia. J Infect 2016; 72:544–53. 26916042 10.1016/j.jinf.2016.02.003

[9] Liu C, Bayer A, Cosgrove SE, et al Clinical practice guidelines by the Infectious Diseases Society of America for the treatment of methicillin-resistant *Staphylococcus aureus* infections in adults and children. Clin Infect Dis 2011; 52:e18–55. 21208910 10.1093/cid/ciq146

[10] Kuehl R, Morata L, Boeing C, et al Defining persistent *Staphylococcus aureus* bacteraemia: secondary analysis of a prospective cohort study. Lancet Infect Dis 2020; 20:1409–17. 32763194 10.1016/S1473-3099(20)30447-3

[11] Minejima E, Mai N, Bui N, et al Defining the breakpoint duration of *Staphylococcus aureus* bacteremia predictive of poor outcomes. Clin Infect Dis 2020; 70:566–73. 30949675 10.1093/cid/ciz257 PMC7768749

[12] Palraj BR, Baddour LM, Hess EP, et al Predicting Risk of Endocarditis Using a Clinical Tool (PREDICT): scoring system to guide use of echocardiography in the management of *Staphylococcus aureus* bacteremia. Clin Infect Dis 2015; 61:18–28. 25810284 10.1093/cid/civ235 PMC4542912

[13] Simos PA, Holland DJ, Stewart A, et al Clinical prediction scores and the utility of time to blood culture positivity in stratifying the risk of infective endocarditis in *Staphylococcus aureus* bacteraemia. J Antimicrob Chemother 2022; 77:2003–10. 35425988 10.1093/jac/dkac129

[14] Maillart E, Karmali R, Miendje Deyi VY, Mascart G, Cherifi S. The association between time to positivity and *Staphylococcus aureus* bacteremia in a geriatric population. J Med Microb Diagn 2012; 1:114.

[15] Khatib R, Riederer K, Saeed S, et al Time to positivity in *Staphylococcus aureus* bacteremia: possible correlation with the source and outcome of infection. Clin Infect Dis 2005; 41:594–8. 16080079 10.1086/432472

[16] Kahn F, Resman F, Bergmark S, et al Time to blood culture positivity in *Staphylococcus aureus* bacteraemia to determine risk of infective endocarditis. Clin Microbiol Infect 2021; 27:1345.e7–12. 10.1016/j.cmi.2020.11.007 33197608

[17] Nieman AE, Rozemeijer W, Savelkoul PHM, Schade RP. Bacterial DNA load in *Staphylococcus aureus* bacteremia is significantly higher in intravascular infections. PLoS One 2022; 17:e0266869. 35443013 10.1371/journal.pone.0266869 PMC9020692

[18] Guimaraes AO, Gutierrez J, Maskarinec SA, et al Prognostic power of pathogen cell-free DNA in *Staphylococcus aureus* bacteremia. Open Forum Infect Dis 2019; 6:ofz126. 31041341 10.1093/ofid/ofz126 PMC6483138

[19] Siméon S, Le Moing V, Tubiana S, et al Time to blood culture positivity: an independent predictor of infective endocarditis and mortality in patients with *Staphylococcus aureus* bacteraemia. Clin Microbiol Infect 2019; 25:481–8. 30036664 10.1016/j.cmi.2018.07.015

[20] Holland TL, Arnold C, Fowler VG Jr. Clinical management of *Staphylococcus aureus* bacteremia: a review. JAMA 2014; 312:1330–41. 25268440 10.1001/jama.2014.9743 PMC4263314

[21] Holland TL . Early oral antibiotic switch for *Staphylococcus aureus* bacteremia: many are called, but few are chosen. Antimicrob Agents Chemother 2020; 64:e00317-20. 32393495 10.1128/AAC.00317-20 PMC7317999

[22] Kaasch AJ, López-Cortés LE, Rodríguez-Baño J, et al Efficacy and safety of an early oral switch in low-risk *Staphylococcus aureus* bloodstream infection (SABATO): an international, open-label, parallel-group, randomised, controlled, non-inferiority trial. Lancet Infect Dis 2024; 24:523–34. 38244557 10.1016/S1473-3099(23)00756-9

[23] Willekens R, Puig-Asensio M, Ruiz-Camps I, et al Early oral switch to linezolid for low-risk patients with *Staphylococcus aureus* bloodstream infections: a propensity-matched cohort study. Clin Infect Dis 2019; 69:381–7. 30351401 10.1093/cid/ciy916

[24] Bupha-Intr O, Blackmore T, Bloomfield M. Efficacy of early oral switch with β-lactams for low-risk *Staphylococcus aureus* bacteremia. Antimicrob Agents Chemother 2020; 64:e02345-19. 32015029 10.1128/AAC.02345-19 PMC7318028

[25] Charlson ME, Pompei P, Ales KL, MacKenzie CR. A new method of classifying prognostic comorbidity in longitudinal studies: development and validation. J Chronic Dis 1987; 40:373–83. 3558716 10.1016/0021-9681(87)90171-8

[26] Li JS, Sexton DJ, Mick N, et al Proposed modifications to the Duke criteria for the diagnosis of infective endocarditis. Clin Infect Dis 2000; 30:633–8. 10770721 10.1086/313753

[27] Friedman ND, Kaye KS, Stout JE, et al Health care—associated bloodstream infections in adults: a reason to change the accepted definition of community-acquired infections. Ann Intern Med 2002; 137:791–7. 12435215 10.7326/0003-4819-137-10-200211190-00007

[28] Martínez JA, Pozo L, Almela M, et al Microbial and clinical determinants of time-to-positivity in patients with bacteraemia. Clin Microbiol Infect 2007; 13:709–16. 17484763 10.1111/j.1469-0691.2007.01736.x

[29] Bai AD, Agarwal A, Steinberg M, et al Clinical predictors and clinical prediction rules to estimate initial patient risk for infective endocarditis in *Staphylococcus aureus* bacteraemia: a systematic review and meta-analysis. Clin Microbiol Infect 2017; 23:900–6. 28487168 10.1016/j.cmi.2017.04.025

[30] Chong YP, Park S-J, Kim HS, et al Persistent *Staphylococcus aureus* bacteremia: a prospective analysis of risk factors, outcomes, and microbiologic and genotypic characteristics of isolates. Medicine (Baltimore) 2013; 92:98–108. 23429353 10.1097/MD.0b013e318289ff1e PMC4553980

[31] Thorlacius-Ussing L, Sandholdt H, Nissen J, et al Comparable outcomes of short-course and prolonged-course therapy in selected cases of methicillin-susceptible *Staphylococcus aureus* bacteremia: a pooled cohort study. Clin Infect Dis 2021; 73:866–72. 33677515 10.1093/cid/ciab201

[32] Kaasch AJ, Barlow G, Edgeworth JD, et al *Staphylococcus aureus* bloodstream infection: a pooled analysis of five prospective, observational studies. J Infect 2014; 68:242–51. 24247070 10.1016/j.jinf.2013.10.015 PMC4136490

