# Supplementary material for: Time to Staphylococcus aureus Blood Culture Positivity as a Risk Marker of Infective Endocarditis: A Retrospective Cohort Study
Source: Clin Infect Dis. 2024 Dec 21;80(4):727–34. doi: 10.1093/cid/ciae628 (PMC12043066; doi:10.1093/cid/ciae628)
Supplement: ciae628_Supplementary_Data [file ciae628_supplementary_data.docx]

Supplementary table 1. Time to positivity to exclude endocarditis in the whole cohort, the community acquired group and community-onset group before and after immediate incubation of blood culture flasks was introduced in 2018.

|  | | 13-hour cut-off (median) | | | 17-hour cut-off (75^th^ percentile) | | |
| --- | --- | --- | --- | --- | --- | --- | --- |
|  |  | Community acquired | Community-onset | Whole cohort | Community acquired | Community-onset | Whole cohort |
| 2011–2021 | | | | | | | |
|  | Number (%) over cut-off | 245/502 (49) | 612/1199 (51) | 858/1702 (50) | 118/502 (24) | 309/1199 (26) | 428/1702 (25) |
|  | IE missed (%) | 8/68 (12) | 22/122 (18) | 34/154 (22) | 4/68 (6) | 10/122 (8) | 16/154 (10) |
|  | Negative predictive value (95% CI) | 97% (93-98) | 96% (95-98) | 96% (95-97) | 97% (92-99) | 97% (94-98) | 96% (94-98) |
| 2019–2021 | | | | | | | |
|  | Number (%) over cut-off | 113/212 (53) | 239/452 (53) | 354/674 (53) | 48/212 (23) | 102/452 (23) | 149/674 (22) |
|  | IE missed (%) | 2/29 (7) | 5/46 (11) | 10/58 (17) | 0/29 | 1/46 (2) | 3/58 (5) |
|  | Negative predictive value (95% CI) | 97% (90-99) | 98% (95-99) | 97% (95-98) | 100% (93-100) | 99% (94-100) | 98%(94-99) |

Supplementary table 2. Presence of pacemaker (PM), implantable cardioverter-defibrillator (ICD) or prosthetic valves in endocarditis cases with time-to-positivity above cut-off in the whole cohort.

|  | 13-hour cut-off | | 17-hour cut-off | |
| --- | --- | --- | --- | --- |
| Time period | **2011-2021** | **2019-2021** | **2011-2021** | **2019-2021** |
| PM and/or ICD (%) | 7/34 (21) | 3/10 (30) | 4/16 (25) | 1/3 (33) |
| Prosthetic valves (%) | 2/34 (6) | 2/10 (20) | 1/16 (6) | 1/3 (33) |
